# Association between Polymorphisms in Antioxidant Genes and Inflammatory Bowel Disease

**DOI:** 10.1371/journal.pone.0169102

**Published:** 2017-01-04

**Authors:** Cristiana Costa Pereira, Cecília Durães, Rosa Coelho, Daniela Grácio, Marco Silva, Armando Peixoto, Paula Lago, Márcia Pereira, Telmo Catarino, Salomé Pinho, João Paulo Teixeira, Guilherme Macedo, Vito Annese, Fernando Magro

**Affiliations:** 1 Department of Environmental Health, National Institute of Health Dr. Ricardo Jorge, Oporto, Portugal; 2 MedInUP – Centre for Drug Discovery and Innovative Medicines, University of Porto, Oporto, Portugal; 3 EPIUnit – Institute of Public Health, University of Porto, Oporto, Portugal; 4 Ipatimup – Institute of Molecular Pathology and Immunology of the University of Porto, Oporto, Portugal; 5 i3S – Instituto de Investigação e Inovação em Saúde, Universidade do Porto, Oporto, Portugal; 6 Department of Gastroenterology, Faculty of Medicine, Centro Hospitalar São João, Oporto, Portugal; 7 Department of Pharmacology and Therapeutics, Faculty of Medicine, University of Porto, Oporto, Portugal; 8 Department of Gastroenterology, HSA – Centro Hospitalar do Porto, Oporto, Portugal; 9 Institute for the Biomedical Sciences Abel Salazar, University of Porto, Oporto, Portugal; 10 Emergency Department, Gastroenterology Unit, AOU Careggi, Florence, Italy; University Hospital Llandough, UNITED KINGDOM

## Abstract

Inflammation is the driving force in inflammatory bowel disease (IBD) and its link to oxidative stress and carcinogenesis has long been accepted. The antioxidant system of the intestinal mucosa in IBD is compromised resulting in increased oxidative injury. This defective antioxidant system may be the result of genetic variants in antioxidant genes, which can represent susceptibility factors for IBD, namely Crohn’s disease (CD) and ulcerative colitis (UC). Single nucleotide polymorphisms (SNPs) in the antioxidant genes *SOD2* (rs4880) and *GPX1* (rs1050450) were genotyped in a Portuguese population comprising 436 Crohn’s disease and 367 ulcerative colitis patients, and 434 healthy controls. We found that the AA genotype in *GPX1* is associated with ulcerative colitis (OR = 1.93, adjusted *P-value* = 0.037). Moreover, we found nominal significant associations between *SOD2* and Crohn’s disease susceptibility and disease subphenotypes but these did not withstand the correction for multiple testing. These findings indicate a possible link between disease phenotypes and antioxidant genes. These results suggest a potential role for antioxidant genes in IBD pathogenesis and should be considered in future association studies.

## Introduction

Crohn’s disease (CD) and ulcerative colitis (UC) are chronic inflammatory bowel diseases (IBD) characterized by inflammation of the intestinal mucosa triggered by the action of environmental factors in genetically predisposed individuals [1]. The accepted notion is that the two major classifications of inflammatory bowel disease (IBD), known as Crohn's disease (CD) and ulcerative colitis (UC), are indeed distinct entities and have different causes and discrete mechanisms of tissue inflammation and damage. UC results in inflammation and ulcerations in the mucosal lining of the colon and rectum [2–6]. Crohn’s disease differs in that it may result in inflammation deeper within the intestinal wall (transmural) and can occur in any parts of the digestive system (the mouth, esophagus, stomach, duodenum, small intestine, colon and rectum). Further, CD may also involve other organs outside the GI system through fistulization [7, 8].

Reactive oxygen species (ROS) have been suggested as key molecules in mediating the tissue injuries promoted by the inflammatory processes occurring in IBD [9, 10], and oxidative stress has been recognized as a potential etiological factor for IBD [11]. The detrimental effects of oxidative stress may be promoted and/or exacerbated by impairments of cellular antioxidant systems. The activation of inflammatory cells and consequent oxidative stress are mechanisms that have already been associated with carcinogenic processes, with 25% of all cancer cases worldwide attributed to chronic inflammation [12]. Recent reports refer 1.9- and 2.4-fold increased risk for CRC among CD and UC patients, respectively [13].

The antioxidant defence systems protect cells against ROS by regulating their intracellular concentrations through the activity of a number of enzymes, including superoxide dismutase 2 (SOD2) and glutathione peroxidase 1 (GPX1). These two enzymes integrate a common detoxification pathway, in which SOD2 (a tetrameric manganese-containing enzyme expressed in mitochondria) first catalyses the dismutation of superoxide anion to hydrogen peroxide and oxygen. The GPX1 enzyme (a cytosolic and ubiquous selenoenzyme) catalyses the subsequent conversion of hydrogen peroxide to water and oxygen [14]. Their combined action promotes the detoxification of mitochondrial ROS and a balance is expected to exist between these two enzymes, as a deranged activity would result in the accumulation of toxic levels of hydrogen peroxide in the cells [15]. Genetic polymorphisms can modify the activity of these critical enzymes and thus promote imbalances in the cellular oxidative burden. Diseases such as diabetic nephropathy, cardiomyopathy, Behcet’s disease, and various cancers have already been associated with polymorphisms in antioxidant genes [16]. Genetic polymorphisms in *SOD2* have also been referred [17] as a potential mechanism interfering in the pharmaco-response of cells exposed to methotrexate (MTX), an immunomodulator commonly prescribed to IBD patients, and an interdependent mechanism of action between *SOD2* and *GPX1* has been suggested, indicating that these genes could also have an important role for therapeutic management.

Genome wide association studies (GWAS) have identified more than 200 genetic risk loci for IBD [18, 19] but only few were associated with clinical phenotype [20, 21]. Association analyses identify 38 susceptibility loci for inflammatory bowel disease and highlight shared genetic risk across populations [19]. Moreover, several genetic polymorphisms have already been linked to IBD etiopathogenesis and to response to therapy [22–24]. Based on the described interactions between inflammation, ROS and oxidative damage, this study aimed to assess the association between IBD (CD and UC) and polymorphisms in the antioxidant genes *SOD2* (rs4880, c.47T>C, Val16Ala) and *GPX1* (rs1050450, c.596C>T, Pro198Leu). The association of these polymorphisms with IBD has not been studied before.

## Materials and Methods

### Population

The study comprised 803 IBD patients (436 CD and 367 UC) and 434 controls (Table 1). The control group included 434 unmatched samples obtained from unrelated healthy blood donors (mean age 47.2±18.7 years; female:male ratio: 1.4:1). This group consisted of permanent residents in the catchment area of Hospital of S. João (Porto, Portugal), selected during the assembling of the EpiPorto cohort [25]. Enrollment of participants was performed under approval of Centro Hospitalar S. João ethic committee (Comissão de Ética para a Saúde do Centro Hospitalar S. João) and included written informed consent for data and DNA usage, obtained by trained personnel (nurses and doctors) upon collection. The participants included did not present any apparent infectious and/or chronic disorders.

**Table 1:** Clinical characteristics of patients with Crohn's disease and ulcerative colitis.

Patients characteristics	Crohn’s disease		ulcerative colitis	
	n	%	n	%
Total	436	-	367	-
Family history of IBD (yes/no*)	37/348	9.6/90.4	16/231	6.5/93.5
Sex (male/female)	225/211	51.6/48.4	162/205	44.1/55.9
Smoking habits	-	-	-	-
Never	189	46.2	222	63.8
Former	108	26.4	91	26.2
Current	112	27.4	35	10.1
Age at diagnosis	-	-	-	-
A1 (<17 years)	56	12.8	14	3.8
A2 (17–40 years)	306	70.2	215	58.6
A3 (>40)	74	17.0	138	37.6
Location	436	-	na	-
L1* (ileal)	195	44.8	na	-
L2 (colonic)	60	13.8	na	-
L3 (ilealcolonic)	180	41.4	na	-
L4 (L1_4_+L2_4_+L3_4_)** (involvement of the upper digestive tract)	47	(10.8)	na	-
Location	na	-	364	-
E1* (proctitis + rectosigmoid)	na	-	134	36.8
E2 (distal)	na	-	101	27.8
E3 (pancolitis)	na	-	129	35.4
Behaviour	431	-	na	-
B1* (nonstricturing, nonpenetrating)	187	43.4	na	-
B2 (stricturing)	90	20.9	na	-
B3 (penetrating)	154	35.7	na	-
P (B1_P_+B2_P_+B3_P_)** (perianal disease)	41	(9.5)	na	-
Rectal involvement (yes/no*)	99/337	22.7/77.3	228/8	96.6/3.4
Colonic involvement (yes/no*)	229/207	52.5/47.5	198/38	83.9/16.1
Abdominal surgery (yes/no*)	226/208	52.1/47.9	23/342	6.3/93.7
Extraintestinal manifestations (yes/no*)	53/383	12.2/87.8	7/230	3.0/97.0
Previous ongoing corticosteroids (yes/no*)	398/36	91.7/8.3	225/141	61.5/38.5
Steroid dependency (yes/no*)	189/243	43.8/56.3	81/206	28.2/71.8
Steroid resistance (yes/no*)	11/422	2.5/97.5	26/261	9.1/90.9
Need for immunosuppressant (yes/no*)	359/50	87.8/12.2	130/236	35.5/64.5
Response to immunosuppressant (yes*/no)	229/138	62.4/37.6	72/51	58.5/41.5
Response to biologics (yes*/no)	198/42	82.5/17.5	50/19	72.5/27.5

* Represents the reference in the case-case association studies;

** For L4 and P phenotypes the reference is absence of phenotype;

na: not applicable

The IBD group included 436 CD and 237 UC patients enrolled at Centro Hospitalar São João, Porto, Portugal; these patients attended their routine IBD specialist medical appointment and all were prospectively followed-up in a national database from Portuguese IBD group (GEDII, https://gediibasedados.med.up.pt/). An additional 130 UC patients were enrolled at Centro Hospitalar do Porto, Hospital de Santo António (CHP-HSA), Porto, Portugal, prospectively followed at the same hospital. The diagnosis of IBD was made according to the ECCO (European Crohn’s and Colitis Organisation) guidelines for CD [26] and UC [27] diagnosis, and the Lennard-Jones criteria [28]. Information on patients’ characteristics was obtained, namely smoking habits, age at diagnosis, years of follow-up, location and behaviour of disease, extra-intestinal manifestations, rectal and colonic involvement, previous abdominal surgery, previous corticotherapy, steroid dependency and resistance, need for immunosuppressant, response to immunosuppressant and response to biologic therapy (Table 1). Location, behaviour and age at diagnosis, were classified according to the Montreal Classification [29]. Patients were defined as steroid-dependent when incapable to reduce steroids below the equivalent of prednisolone of 10mg per day within three months of starting steroids without recurrent active disease or disease relapse within three months of stopping steroids. The steroid resistance was considered as the presence of active disease despite of a prednisolone dose of up to 0.75mg/kg per day over a period of four weeks [30]. Patients who had treatment with azathioprine, or methotrexate were considered in the group of ‘need for immunosuppressant’. The ‘response to immunosuppressant’ was defined as positive when long-term sustained improvement of the symptoms was achieved, lasting at least one year without any further modifications in the therapeutic regime; the response was considered negative whenever no symptom improvement was verified after three months of full dose with azathioprine or methotrexate or by decision of the physician to add steroids or biologic therapy (anti-TNFα) or to refer for surgery. The ‘response to biologic therapy’ was defined as positive when long-term sustained improvement of the symptoms lasting at least one year without any further modifications in the therapeutic regimen was observed; failure of the therapeutic regimen was defined by an absence of improvement of the symptoms of disease and by decision of the physician to add steroids, add an immunosuppressant, switch to an alternative biologic therapy medication or to refer for surgery. S1 Table shows the clinical characteristics defining the phenotypes tested for association (reference categories used in the association analyses are marked with an asterisk). The Ethics Committee of both institutions—Comissão de Ética para a Saúde do Centro Hospitalar São João, and Comissão de Ética para a Saúde do Centro Hospitalar do Porto—approved the protocol and all patients or their legal guardians gave their written informed consent, complying with the principles laid down in the Declaration of Helsinki.

### SNP selection and genotyping

We identified two potential functional polymorphisms involved in antioxidant pathways–*SOD2* A/G (rs4880) and *GPX1* G/A (rs1050450)–whose main effects have already been described for other conditions. These SNPs have a reported minor allele frequency of ≥ 0.1 for the European Caucasian population (S2 Table). Patients’ and controls’ genomic DNA was isolated from blood using the QIAcube system and the QIAamp DNA Blood Mini QIAcube Kit (Qiagen, Venlo, The Netherlands) following the supplier's instructions. The SNPs were genotyped on the complementary DNA strand using TaqMan Pre-Designed SNP Genotyping Assay (Life Technologies, Carlsbad, CA, USA) (S3 Table). DNA amplification and allelic discrimination were performed according to product specifications with the ABI 7500 Fast real-time PCR system (Applied Biosystems, Carlsbad, USA). Cases and controls were randomized during genotyping and 5% of the samples were genotyped in duplicate to assess the genotyping error rate. Concordance of genotypes was 100%.

### Statistical analysis

Genotyping results were evaluated with the SNPassoc 1.6–0 package in the statistical software suite R. Compliance of alleles at individual loci with Hardy-Weinberg equilibrium was measured at the level of the control group using a χ^2^ test (P-value<0.05). Power calculations conducted before the study indicated there was more than 80% power to detect significant associations of OR between 1.3 and 2.0 for both SNPs and both disease groups. Calculations were performed using CaTS software with the following settings: *SOD2* (rs4880): MAF = 0.47, OR = 1.25–2.00, CD and UC prevalence Portugal = 0.07% [31], CD case/control = 340/330, UC case/control = 370/330; *GPX1* (rs1050450): MAF = 0.34, OR = 1.25–2.00, CD and UC prevalence Portugal = 0.07%[31], case/control = 340/330, UC case/control = 370/330. A post-hoc power analysis showed that the power to detect significant associations is 100% for both disease groups since the ORs observed fall within the 1.5–2.0 range (or the equivalent 0.5–1.0 range).

Comparison of genotype frequencies between groups defined by status (patients vs. controls) and clinical characteristics were assessed by unconditional logistic regression (level of significance set to P-value<0.05) using the SNPassoc library in R and SPSS 23 (IBM SPSS statistics). The models included adjustment by sex and age. The codominant and recessive models of inheritance were considered. Odds ratios (OR) with respective confidence intervals (95% CI) were calculated for the allele (one copy or genotype) with minor frequency. The association of SNPs with CD and UC clinical characteristics was subsequently assessed using case-case analyses (S3 Table). The IBD phenotypes and reference categories are defined in Table 1. The Bonferroni correction was used to adjust for multiple testing in the analysis of overall association of SNPs with CD and UC (Table 2) and in the case-case analysis (Table 3). The correction was applied separately to each disease group.

**Table 2:** Genotypic frequencies and overall association of genetic variants in *SOD2* and *GPX1* with Crohn’s disease and ulcerative colitis.

Locus	Model	Controls n = 434	Crohn’s disease n = 436	OR (95% CI)	P-value	P-value adjusted	Ulcerative colitis n = 367	OR (95% CI)	P-value	P-value adjusted
*SOD2* rs4880		n = 426	n = 435				n = 367			
	AA*	119 (27.9)	142 (32.6)	1.00			102 (27.8)	1.00		
	GA	198 (46.5)	214 (49.2)	0.79 (0.55–1.13)	0.193	1.00	184 (50.1)	1.07 (0.75–1.51)	0.700	1.00
	GG	109 (25.6)	79 (18.2)	0.57 (0.37–0.89)	0.013	0.077	81 (22.1)	0.89 (0.59–1.34)	0.547	1.00
	A carrier* *vs*. GG	317 (74.4)/109 (25.6)	356 (81.8)/79 (18.2)	0.66 (0.45–0.97)	0.033	0.197	286 (77.9)/81 (22.1)	0.85 (0.60–1.20)	0.366	1.00
*GPX1* rs1050450		n = 428	n = 430				n = 367			
	GG*	199 (46.5)	191 (44.4)	1.00			146 (39.8)	1.00		
	GA	187 (43.7)	187 (43.5)	0.96 (0.69–1.34)	0.802	1.00	164 (44.7)	1.18 (0.86–1.61)	0.277	1.00
	AA	42 (9.8)	52 (12.1)	1.39 (0.80–2.40)	0.242	1.00	57 (15.5)	**1.93 (1.20–3.12)**	**0.006**	0.037
	G carrier* *vs*. AA	386 (90.2)/42 (9.8)	378 (87.9)/52 (12.1)	1.39 (0.83–1.38)	0.210	1.00	310 (84.5)/57 (15.5)	**1.78 (1.13–2.80)**	**0.012**	0.070

* Reference;

ORs and 95% CIs were calculated considering the codominant and recessive models, adjusted for gender and age; bold font indicates nominally significant results; **p-value cutoff = 0.0083** (after Bonferroni correction applied separately to each disease group); SNPs were genotyped on the complementary DNA strand.

**Table 3:** Association of SNPs in the antioxidant system genes *SOD2* and *GPX1* with Crohn’s disease clinical characteristics.

Locus		Ilealcolonic (L3)**		Rectal involvement		Colonic involvement		Responds to biologics	
	**SNP / Model**	OR	*P*-value	OR	*P*-value	OR	*P*-value	OR	*P*-value
		(95% CI)		(95% CI)		(95% CI)		(95% CI)	
*SOD2* rs4880	**AA**	1.00		1.00		1.00		1.00	
	**GA**	0.82	0.577	1.23	0.457	0.84	0.442	1.48	0.347
		(0.41–1.65)		(0.72–2.10)		(0.55–1.30)		(0.65–3.35)	
	**GG**	0.71	0.422	**2.08**	**0.026**	1.74	0.059	1.08	0.890
		(0.31–1.63)		**(1.09–3.96)**		(0.98–3.10)		(0.37–3.10)	
	A carrier* **vs. GG**	0.80	0.546	**1.83**	**0.034**	**1.93**	**0.011**	0.83	0.682
		(0.40–1.63)		**(1.06–3.16)**		**(1.15–3.22)**		(0.34–2.03)	
*GPX1*rs1050450	GG*	1.00		1.00		1.00		1.00	
	**GA**	**2.70**	**0.003**	1.01	0.965	0.86	0.132	1.57	0.26
		**(1.39–5.24)**		(0.62–1.63)		(0.57–1.30)		(0.72–3.43)	
	**AA**	1.81	0.250	0.61	0.093	0.62	0.475	**3.10**	**0.032**
		(0.66–4.95)		(0.26–1.40)		(0.33–1.16)		**(1.10–8.70)**	
	G carrier* **vs. AA**	1.17	0.750	0.60	0.196	0.66	0.175	2.42	0.071
		(0.44–3.10)		(0.27–1.34)		(0.37–1.20)		(0.96–6.10)	

* Reference; phenotype reference categories were defined as in Table 1;

** Reference category is “colonic” location;

ORs and 95% CIs were calculated considering the recessive and codominant models, adjusted for gender and age;

Bold font indicates nominally significant results;

**P-value cutoff = 0.00063** (after Bonferroni correction applied to 80 tests performed in the case-case analysis in the CD group);

SNPs were genotyped on the complementary DNA strand.

## Results

### Genotypic frequencies and overall association with CD and UC

We genotyped two SNPs in antioxidant enzyme genes (*SOD2* and *GPX1*). In the control group, the frequencies of all SNPs did not deviate significantly from those expected under Hardy-Weinberg equilibrium (*P*>0.05). Table 2 summarizes the genotype frequencies for the SNPs rs4880 in *SOD2* and rs1050450 in *GPX1*, and overall associations with CD and UC (OR, 95% CI and respective *P*-values).

Based on the two models analysed—codominant and recessive—SNP rs1050450 in *GPX1* achieved nominal significant association with UC with *P*-values of 0.006, in the codominant, and 0.012, for the recessive model. In the codominant model the homozygous AA showed an OR (CI) of 1.93 (1.20–3.12) and in the recessive model an OR (CI) of 1.78 (1.13–2.80) is observed. After correction for multiple testing (Bonferroni correction) the homozygous AA genotype of *GPX1* (rs1050450) withstood the association with UC with an adjusted P-value of 0.037. SNP rs4880 in *SOD2* achieved a nominal significant association with CD in both models analysed, codominant and recessive, with *P*-values of 0.013 and 0.033, respectively. The homozygous GG showed an OR (CI) of 0.57 (0.37–0.89) in the codominant model and an OR (CI) of 0.66 (0.45–0.97) in the recessive model. These associations were lost when corrected for multiple testing. The SNPs were also considered for analysis with CD and UC phenotypes (genotype frequencies are reported in S4 and S5 Tables, respectively).

Bonferroni correction for the genotype-phenotype case-case analyses in CD established a p-value of 0.00063 (Table 3), and none of the associations found could remain significant. Nevertheless, nominal p-values were found significant for some phenotypes, as follows:

### Genotype association with CD phenotypes

Under the recessive model (Table 3), we found significant associations between SNP rs4880 in *SOD2* and ‘rectal involvement’ [OR (CI) = 1.83 (1.03–1.98), *P* = 0.034], and ‘colonic involvement’ [OR(CI) 1.93 (1.15–3.22), *P* = 0.011]. In the codominant model (Table 3), *SOD2* homozygous GG was associated with ‘rectal involvement’ [OR (CI) = 2.08 (1.09–3.96), *P* = 0.026]. *GPX1* homozygous AA associated with ‘response to biologics’ [OR (CI) = 3.10 (1.10–8.70), *P* = 0.032], and the heterozygous GA genotype was found significantly associated with ‘location (L3 vs L2)’ [OR (CI) = 2.70 (1.39–5.24), *P* = 0.003],

### Genotype association with UC phenotypes

Within UC, under the recessive model we found significant associations between SNP rs4880 in SOD2 and location (E2 vs E1) [OR (CI) = 0.50 (0.26–0.96), *P* = 0.032] (data not shown in table).

### Combined genotypes and risk profiles

Joint effects of the risk genotypes observed for CD and UC phenotypes have been assessed, however, no significant associations were found (*P*>0.05 for all the combinations tested; data not shown).

## Discussion

The characterization of susceptibility genes in IBD is expected to bring benefit for the identification of primary pathogenic pathways, and possible environmental drivers, as well as new therapeutic targets. To clarify whether polymorphisms in antioxidant enzyme genes were associated with IBD (CD and UC) we conducted case-control and case-case studies for two SNPs in antioxidant genes (*SOD2* Val16Ala and *GPX1* Pro198Leu). To our best knowledge, this is the first study investigating the association between these genetic variants and IBD pathogenesis. Among the already published GWAS studies in IBD the only reference found related to these two genes is for GPX1 that has been suggested [32] to be in linkage disequilibrium with macrophage stimulating protein-1 (MST1); the authors propose that SNP rs1050450 (c.596C>T) in *GPX1* is the pathophysiologic link between *IBD12* locus and IBD, rather than the macrophage stimulating protein-1 (*MST1*), as previously described.

In our study the allele A in *GPX1* (rs1050450) significantly associated with UC in the recessive model with an ORs of 1.93, and an adjusted *P*-value of 0.037. Antioxidant enzymes maintain cellular redox homeostasis. Glutathione peroxidase (GPX) is a selenoenzyme that catalyses the breakdown of hydrogen peroxide (H_2_O_2_), and other organic peroxides, into water (H_2_O) and oxygen (O_2_). GPX1 is cytosolic and produced in all tissues. The SNP rs1050450 (or *GPX1* Pro198Leu) has been studied extensively in human disease and has already been linked to cancer risk [33], and oxidative stress related diseases [34]. The Leu198 variant results in a 10% reduced enzyme activity compared with the Pro198 variant [35], interfering with the overall capacity to respond to oxidative damage [34]. Individuals with reduced GPX1 activity exhibit an increased incidence of oxidative stress-related diseases such as breast, colon, prostate, bladder and lung cancers, coronary artery disease, and also, low bone mineral density [36] and osteoarthropathy [37].

Studies using genetically altered mice with reduced GPX1 activity have shown a link to chronic and acute gastrointestinal inflammation [38]. In this study, we found that the variant with lower activity *GPX1* 198Leu associates with UC (OR 1.93, *P* = 0.006). In the CD group, a 3.1-fold increased odds for ‘responding to biologics’ was found for carriers of the homozygous variant Leu198Leu (*P* = 0.03). These findings along with the recent observation that SNP rs1050450 in *GPX1* is the pathophysiological link for IBD locus 12 [32], suggest that this gene is a good candidate as a biomarker for disease and treatment management purposes.

The SOD2 enzyme, also known as manganese superoxide dismutase (MnSOD), is one of the major antioxidant defence systems against mitochondrial superoxide radicals [39] and is one of the first in a chain of enzymes to mediate the ROS generated by the partial reduction of O_2_ to hydrogen peroxide (H_2_O_2_). The MnSOD expression has been suggested as a potential biomarker in UC for predicting disease activity and severity [40]. While the valine-containing SOD2 (rs4880) is partially arrested in the inner mitochondrial membrane, the alanine-containing SOD2 (rs4880) is actively targeted to the mitochondrial matrix resulting in a 30–40% increase in SOD2 activity for the Ala16Ala variant, due to more efficient transport of the protein into the mitochondrial matrix [41] and to a 4-fold higher and stable mRNA expression [42]. Crawford et al. (2012) [16] reviewed 79 studies addressing a potential link between the *SOD2* Val16Ala genotype and various diseases or disorders, and almost half of those studies reported relationships with various types of cancers, e.g. gastric, lung, prostate, bladder and breast, to diabetes type I, nephropathy, chronic kidney disease, and to chemotherapy responses. The Ala16Ala genotype is generally associated with a protective effect, yet important differences are described in the literature, which indicates a complex role for the presence of risk allele C. Also, it has been suggested that *SOD2* 16Val variant is associated with an increased production of pro-inflammatory cytokines [43], and that its expression can be modulated through NF-kB binding to the promoter region of the gene [44].

In this study, although significances were lost after correction for multiple testing, nominal significant associations suggest a potential protective role regarding CD. Also, *SOD2* Ala16Ala variant in the CD group presented a 2.08-fold risk for rectal involvement (P = 0.026), and in the recessive model, it also presented 1.93 odds for colonic involvement, These findings might indicate that although this variant is uncommon among CD patients, when present it confers some susceptibility for rectal and colonic involvement. We suggest that overall, this gene might be implicated in disease location, and the fact that the pro-inflammatory prone variant (16Val) is more common among CD, the variant 16Ala is associated with colonic involvement in CD. Also, the referred putative effects of *SOD2* in inflammatory pathways, together with the fact that *SOD2* expression can be induced by dietary intake of antioxidants [45], indicate a potential therapeutic targeting for this gene as well as for dietary intervention benefits.

## Conclusion

This aim of this study was to explore possible associations between IBD pathogenesis (CD and UC) and gene polymorphisms implicated in other oxidative stress conditions, taking into account several clinical characteristics. We found a significant association between the variant *GPX1* (rs1050450) and UC. This association needs to be tested in an independent cohort to validate these findings. Genetic association studies support the idea that disease location is genetically determined and many important loci that could explain disease heterogeneity are still undetermined [20]. Also, the work of Hauser et al. [32] recently published suggesting a pathophysiological role for *GPX1* (rs1050450) is as well suggested with our findings. Overall, our results point out to a potential role of antioxidant genes in IBD pathogenesis.

## Supporting Information

S1 File Dataset. (XLSX)

S1 Table Phenotype categories and number of tests performed for Crohn’s disease and ulcerative colitis case-case analysis (according to the clinical characteristics described in Table 1). (PDF)

S2 Table Characteristics of the SNPs genotyped. (PDF)

S3 Table TaqMan SNP genotyping assay details. (PDF)

S4 Table Summary of genotypic frequencies of SNPs in, *SOD2*, and *GPX1* [n (%)] in the Crohn’s disease group. Cases subdivided according to location, rectal involvement, colonic involvement and response to biologics.
(PDF)

S5 Table Summary of genotypic frequencies of SNPs in, *SOD2*, and *GPX1* [n (%)] in the ulcerative colitis group. Cases subdivided according to ‘location.
(PDF)
