# Supplementary material for: Association between Polymorphisms in Antioxidant Genes and Inflammatory Bowel Disease
Source: PLoS One. 2017 Jan 4;12(1):e0169102. doi: 10.1371/journal.pone.0169102 (PMC5215755; doi:10.1371/journal.pone.0169102)
Supplement: S1 Table — (PDF) [file pone.0169102.s002.pdf]

TABLE S1. Phenotype categories and number of tests performed for Crohn's disease and ulcerative colitis case-case analysis (according to the clinical characteristics described in Table 1)

CROHN'S DISEASE			ULCERATIVE COLITIS		
Test	Phenotype	Categories	Test	Phenotype	Categories
1	Family history of IBD	no* vs yes	1	Family history of IBD	no* vs yes
2	Location	L1* vs L2	2	Location	E1* vs E2
3	Location	L1* vs L3	3	Location	E1* vs E3
4	Location	L2* vs L3	4	Location	E2* vs E3
5	Location (L4)	no* vs yes			
6	Behaviour	B1* vs B2			
7	Behaviour	B1* vs B3			
8	Behaviour	B2* vs B3			
9	Behaviour (P)	no* vs yes			
10	Rectal involvement	no* vs yes	5	Rectal involvement	no* vs yes
11	Colonic involvement	no* vs yes	6	Colonic involvement	no* vs yes
12	Abdominal surgery	no* vs yes	7	Abdominal surgery	no* vs yes
13	Extraintestinal manifestations	no* vs yes	8	Extraintestinal manifestations	no* vs yes
14	Perianal fistulising disease	no* vs yes	9	Perianal fistulising disease	no* vs yes
15	Previous ongoing corticosteroids	no* vs yes	10	Previous ongoing corticosteroids	no* vs yes
16	Steroid dependency	no* vs yes	11	Steroid dependency	no* vs yes
17	Steroid resistance	no* vs yes	12	Steroid resistance	no* vs yes
18	Need for immunosuppressant	no* vs yes	13	Need for immunosuppressant	no* vs yes
19	Response to immunosuppressant	yes* vs no	14	Response to immunosuppressant	yes* vs no
20	Response to biologics	yes* vs no	15	Response to biologics	yes* vs no

*reference category
