# Supplementary material for: Association between Polymorphisms in Antioxidant Genes and Inflammatory Bowel Disease
Source: PLoS One. 2017 Jan 4;12(1):e0169102. doi: 10.1371/journal.pone.0169102 (PMC5215755; doi:10.1371/journal.pone.0169102)
Supplement: S2 Table — (PDF) [file pone.0169102.s003.pdf]

TABLE S2. Characteristics of the SNPs genotyped

Gene	SNP ID	Chromosome position	Gene position	Alleles (major/minor)	MAF Europe*	HWE (p-value)**
GPX1	rs1050450	3:49357401	Exon 2	G/A	0.34*	0.912
SOD2	rs4880	6:159692840	Exon 2	A/G	0.47*	0.074

*data from the 1000 Genomes Project Phase 3

**as measured in this study’s control population
