# Supplementary material for: Association between Polymorphisms in Antioxidant Genes and Inflammatory Bowel Disease
Source: PLoS One. 2017 Jan 4;12(1):e0169102. doi: 10.1371/journal.pone.0169102 (PMC5215755; doi:10.1371/journal.pone.0169102)
Supplement: S3 Table — (PDF) [file pone.0169102.s004.pdf]

TABLE S3. TaqMan SNP genotyping assay details.

Gene	SNP ID	Forward primer	Reverse primer
GPX1	rs1050450*	CACTGCAACTGCCAAGCA	CAGACCATTGACATCGAGCCT
SOD2	rs4880	Not available**	Not available**

*Custom design genotyping assay

**ThermoFisher Scientific does not provide primer sequences for SNP TaqMan assays available on their database
