# Supplementary material for: Association between Polymorphisms in Antioxidant Genes and Inflammatory Bowel Disease
Source: PLoS One. 2017 Jan 4;12(1):e0169102. doi: 10.1371/journal.pone.0169102 (PMC5215755; doi:10.1371/journal.pone.0169102)
Supplement: S4 Table — Cases subdivided according to location, rectal involvement, colonic involvement and response to biologics. (PDF) [file pone.0169102.s005.pdf]

TABLE S4. Summary of genotypic frequencies of SNPs in, *SOD2*, and *GPX1* [n (%)] in the Crohn's disease group. Cases are subdivided according to location, rectal involvement, colonic involvement and response to biologics.

	Location			Rectal involvement		Colonic Involvement		Responds to biologics	
**SNP Genotype**	L1	L2	L3	No	Yes	No	Yes	Yes	No
SOD2	*n*=195	*n=59*	*n=179*	*n*=336	*n*=99	*n*=207	*n*=228	*n*=198	*n*=41
**AA**	66 (33.8)	17 (28.8)	59 (33.0)	116 (34.5)	26 (26.3)	69 (33.3)	73 (32.0)	61 (30.8)	10 (24.4)
**GA**	102 (52.3)	28 (47.5)	83 (46.4)	166 (46.4)	48 (48.5)	110 (53.1)	104 (45.6)	95 (48.0)	24 (58.5)
**GG**	27 (13.8)	14 (23.7)	37 (20.7)	54 (16.1)	25 (25.3)	28 (13.5)	51 (22.4)	42 (21.2)	7 (17.1)
GPX1	*n*=193	*n=59*	*n=176*	*n*=332	*n*=98	*n*=205	*n*=225	*n*=196	*n*=41
**GG**	89 (46.1)	35 (59.3)	67 (38.1)	147 (44.3)	44 (44.9)	87 (42.4)	104 (46.2)	88 (44.9)	12 (29.3)
**GA**	79 (40.9)	18 (30.5)	89 (50.6)	141 (42.5)	46 (46.9)	88 (42.9)	99 (44.0)	88 (44.9)	21 (51.2)
**AA**	25 (13.0)	6 (10.2)	20 (11.4)	44 (13.3)	8 (8.2)	30 (14.6)	22 (9.8)	20 (10.2)	8 (19.5)
