# Supplementary material for: Association between Polymorphisms in Antioxidant Genes and Inflammatory Bowel Disease
Source: PLoS One. 2017 Jan 4;12(1):e0169102. doi: 10.1371/journal.pone.0169102 (PMC5215755; doi:10.1371/journal.pone.0169102)
Supplement: S5 Table — Cases subdivided according to ‘location. (PDF) [file pone.0169102.s006.pdf]

**TABLE S5.** Summary of genotypic frequencies of SNPs in, *SOD2*, and *GPX1* [n (%)] in the ulcerative colitis group. Cases are subdivided according to 'location'.

| | **Location** | | |
|---|---|---|---|
| **SNP Genotype** | E1 | E2 | E3 |
| ***SOD2*** | *n*=134 | *n*=101 | *n*=129 |
| **AA** | 33 (24.6) | 32 (31.7) | 36 (27.9) |
| **GA** | 63 (47.0) | 52 (51.5) | 69 (53.5) |
| **GG** | 38 (28.4) | 17 (16.8) | 24 (18.6) |
| ***GPX1*** | *n*=134 | *n*=101 | *n*=129 |
| **GG** | 50 (37.3) | 44 (43.6) | 51 (39.5) |
| **GA** | 60 (44.8) | 42 (41.6) | 60 (46.5) |
| **AA** | 24 (17.9) | 15 (14.9) | 18 (14.0) |
